# Progression from macular retinoschisis to retinal detachment in highly myopic eyes is associated with outer lamellar hole formation

**DOI:** 10.1136/bjo.2007.131359

**Published:** 2008-06

**Authors:** N Shimada, K Ohno-Matsui, T Yoshida, Y Sugamoto, T Tokoro, M Mochizuki

**Affiliations:** Department of Ophthalmology and Visual Science, Tokyo Medical and Dental University, Tokyo, Japan

## Abstract

**Aims:**

To investigate the morphological changes that occur during the development of an early retinal detachment (RD) from a myopic macular retinoschisis (MRS) by optical coherence tomography (OCT).

**Methods:**

The OCT images of five eyes of five consecutive patients with myopic MRS who developed an RD during the follow-up period were studied.

**Results:**

The progression from MRS to early RDs went through four stages. In stage 1, OCT images appeared to show a focal irregularity of the thickness of external retina. In stage 2, an outer lamellar hole developed within the thickened area and a small RD developed. In stage 3, the column-like structures overlying the hole seemed to be separated horizontally, and the outer lamellar hole appeared to be larger vertically. In stage 4, the upper edge of the external retina was further elevated and attached to the upper part of the retinoschisis layer accompanied by further enlargement of the RD.

**Conclusions:**

This longitudinal study showed that the progression from myopic MRS to RD passes through four stages, and the formation of an outer lamellar hole predisposes the retina to a RD. These OCT findings might be useful for considering the surgical indication for eyes with a myopic MRS.

Macular retinoschisis (MRS) is not uncommon in highly myopic eyes,^1^ ^2^ and some eyes with an MRS progress to a macular hole or a retinal detachment (RD).^3^ ^4^ Traction of the vitreous on the fovea plays a role in the development of a macular hole in eyes with myopic MRS.^3^ ^4^ However, the mechanism causing a RD from a MRS has not been fully clarified. The purpose of this study was to determine the mechanism(s) causing the development of a RD from a MRS. To accomplish this, we examined optical coherence tomographic (OCT) images of five eyes longitudinally, and we shall show that the progression from an MRS to an RD occurred in four stages including the formation of an outer lamellar hole.

## PATIENTS AND METHODS

The medical records of five eyes of five consecutive patients with myopic MRS who developed a RD during the follow-up were examined. The five patients were examined in the High Myopia Clinic of the Tokyo Medical and Dental University from May 2005 to June 2007. Ophthalmoscopic examinations, the best-corrected visual acuity (BCVA) and OCT examinations (OCT Ophthalmoscope, NIDEK, Gamagori, Japan) were performed monthly. If any changes were detected, OCT was performed every 2 weeks. The OCT features were obtained by multiple 8 mm scans centred on the fovea. The thickest value of the macula was measured on the multiple vertically scanned images. The significance of the changes in the thickest value of the macula was determined using a Mann–Whitney U test. A p value of <0.05 was considered significant.

## RESULTS

**Figure 1:**
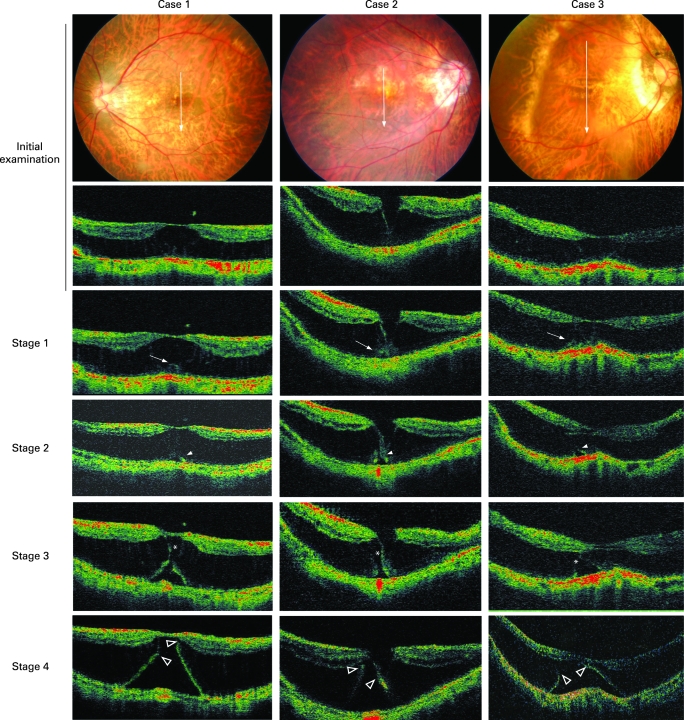
Different stages in the progression from myopic macular retinoschisis to early retinal detachment. Top row: posterior fundus photographs at the initial examination. Second to fifth rows: optical coherence tomographic (OCT) images at the initial examination and at stages 1, 2, 3 and 4. At the initial examination, the OCT images show macular retinoschisis without a retinal detachment. Only Case 2 has an outer lamellar hole. The outer retinal layer appears to be normal. At stage 1, the OCT images show a focal thickening of the outer retinal layers (arrow), and at stage 2, a lamellar hole (arrowhead) is present beneath the thickened area. At stage 3, the retinoschisis layer overlying the outer lamellar hole is separated horizontally (asterisks), and the outer lamellar hole appears enlarged. At stage 4, the upper edge of the external retina (open arrowhead) is attached to the upper part of retinoschisis layer. The RD is larger and is accompanied by the resolution of the retinoschisis.

The clinical characteristics of the five eyes are shown in table 1. Initially, the OCT images showed an MRS without an RD in all eyes (fig 1), but one eye had an inner lamellar hole (case 2, fig 1). The outer retinal layers were split, but there appeared no obvious irregularity of the thickness of the external retina. Also, no vitreous traction on the fovea was detected.

**Table 1:** Characteristics of patients with RD development from macular retinoschisis

No.	Gender	Age	R/L	Refractive error (D)	Axial length (mm)	BCVA					Thickest value of the macula (μm)					Duration from stage 1 to stage 3 (months)	Follow-up (months)
						Initial	Stage 1	Stage 2	Stage 3	Stage 4	Initial	Stage 1	Stage 2	Stage 3	Stage 4		
1	F	56	L	−14.0	29.2	0.8	0.8	0.8	0.7	0.5	474	570	611	782	845	4.5	11
2	F	69	R	−10.0	27.9	0.9	0.8	0.9	0.7	0.4	488	550	561	617	765	4.5	10
3	F	70	R	−15.0	28.6	0.5	0.4	0.5	0.4	0.3	390	390	395	554	876	3.5	17
4	M	46	L	−16.0	29.6	0.9	0.8	0.7	0.7		365	419	440	574		3.5	23
5	F	48	L	−15.5	30.4	1.0	1.0	0.8	0.8	?	440	445	448	574	?	6.5	15

BCVA, best-corrected visual acuity; RD, retinal detachment.

The progression from an MRS to an RD was through four stages (fig 1). In stage 1, the OCT images showed an irregularity of the thickeness of the external retinal layer (arrow), and the focal area of the external retina appeared to be elevated. In stage 2, an outer lamellar hole has developed in the thickened area of the external retina either at the fovea (cases 1, 2, 4) or extrafoveally (cases 3, 5). A small RD is observed beneath the lamellar hole at this stage.

In stage 3, the column-like structures within the retinoschisis layer overlying the lamellar hole appeared to be separated horizontally, and consequently the hole is increased vertically. The outer margins of the external retinal layer around the hole are further elevated, and the area of the RD is enlarged.

In stage 4, the upper edge of external retina around the hole is attached to the upper part of the retinoschisis layer, and RD is further enlarged accompanied by a resolution of the retinoschisis.

A significant decrease in the BCVA (>0.2 logMAR) was observed in three eyes only at stage 4, and all eyes showed a slight but not significant decrease between stages 1 and 3 (table 1). The mean duration from stage 1 to stage 3 was 4.5 (SD 1.2) months (range, 3.5∼6.5 months). The mean thickest value of the macula at stage 3 was 620 (93) μm which is significantly thicker than at the initial examination of 431 (53) μm; p* = *0.01; table 1).

Core vitrectomy, vitreous cortex removal, internal limiting membrane peeling with indocyanine green staining, and gas tamponade was performed on two eyes (cases 4, 5) at stage 3 and 3 eyes (cases 1, 2, 3) at stage 4. In all eyes, the RD disappeared without any intra- or postoperative complications, and the improvement of vision was detected in eyes with stage 3 and stage 4 similarly, although the postoperative follow-up period was short (1∼6 months).

## DISCUSSION

Vitrectomy has been performed on eyes with myopic MRS with or without a RD with good results.^4^ The majority of eyes with a MRS without an RD retain relatively good vision, but once an MRS progresses to a foveal RD, vision usually declines.^4^ We are not certain what the indications are and when the optimal time is for surgery especially for myopic MRS without a RD.

In our five patients, the OCT images showed an irregularity of the thickness of the external retina, and some areas appeared to be elevated at stage 1. Earlier studies showed that an inward traction on the inner retina and the inflexibility of the retinal vessel and rigidity of internal limiting membrane were factors causing an MRS.^5^ Thus, we assume the possibility that an irregularity of the thickeness of the external retinal layer might represent the effects of the traction on the outer retinal layer secondary to an increased inward traction transmitted to the outer retina through the column-like structures within the retinoschisis layer.

An important feature of stage 2 was the development of an outer lamellar hole beneath the thickened retina. A Medline search did not extract any articles reporting on the development of a lamellar hole in the progression of an MRS to RD. We suggest that the retina that is elevated is probably thinner and more fragile, and when the inward traction reaches a critical pressure, a lamellar hole might develop.

In stage 3, the outer lamellar hole has expanded in the vertical direction probably accompanied by an increased separation of the column-like structures within the retinoschisis layer. After that, a vertical shrinkage of the column-like structures might occur, and this would direct the flow of the fluid within the retinoschisis layer into the subretinal space through the hole. We suggest that this flow might continue until the upper edge of the external retina around the hole becomes attached to the upper part of the retinoschisis layer. After this attachment (stage 4), the retinoschsis is resolved, and a further enlargement of the RD occurs as suggested earlier.^6^

The interval from stage 1 to 3 was relatively short (mean; 4.5 months) indicating that we should be very cautious about the progression to RD when the findings in stage 1 are observed in OCT images. Once the lamellar hole develops, an RD will develop in a short time.

In conclusion, our results have shown that an irregularity of the thickeness of the external retina and subsequent formation of outer lamellar hole were present before the RD develops. Thus, these findings are helpful to consider the optical time point of vitreous surgery against myopic MRS.
